# Effect of serum cytokines and VEGF levels on diabetic retinopathy and macular thickness

**Published:** 2009-09-19

**Authors:** Banu Turgut Ozturk, Banu Bozkurt, Hurkan Kerimoglu, Mehmet Okka, Umit Kamis, Kemal Gunduz

**Affiliations:** Department of Ophthalmology, Meram Faculty of Medicine, Selcuk University, Konya, Turkey

## Abstract

**Purpose:**

To investigate the role of serum inflammatory cytokines and vascular endothelial growth factor (VEGF) in diabetic retinopathy (DR) and evaluate their relationship with macular thickness measurements obtained with optical coherence tomography (OCT).

**Methods:**

The study enrolled 28 healthy subjects (Group 1), 31 patients without DR (Group 2), 49 patients with nonproliferative DR (Group 3), and 46 patients with proliferative DR (Group 4). Macular profile was assessed with Stratus OCT-3 and the serum concentrations of VEGF and interleukin-1α (IL-1α), interleukin-6 (IL-6), interleukin-8 (IL-8), interleukin-10 (IL-10), macrophage inflammatory protein (MIP-1α), monocyte chemoattractant protein (MCP-1), and epidermal growth factor (EGF) were measured using multiplex bead immunoassay.

**Results:**

The median value of the visual acuity was 20/20 (Groups 1 and 2), and 20/100 (Group 3), and 20/125 (Group 4). The median value of central subfield macular thickness was estimated as 165.50 μm in Group 1, 202.5 μm in Group 2, 318 μm in Group 3, and 310 μm in Group 4. The median serum VEGF level, which was 98.20 pg/ml in Group 1, demonstrated a progressive rise to 125.37 pg/ml in Group 2, to 153.07 pg/ml in Group 3, and to 149.12 pg/ml in Group 4. Statistical significance was found between all groups (p<0.05) except between Groups 3 and 4 (p=0.87). The median levels of IL-1α and IL-6 were zero in all groups. The median serum levels of IL-8, IL-10, MIP-1α, and EGF revealed a wide range within each group but no statistical significance between the groups (p>0.05). The median serum levels of IL-8, IL-10, MIP-1α, and EGF revealed a wide range within each group, however, no statistically significant relationship was found between the groups (p>0.05). The median values of the serum MCP-1 concentrations presented a statistically significant rise with the progression of DR (p=0.02). No correlation was found between macular thickness and serum cytokine and VEGF levels (p>0.05).

**Conclusions:**

Increased serum levels of VEGF and MCP-1 may act as a key regulator of DR and provide a potential tool for risk assessment in diabetic patients. Further studies that evaluate both vitreous and serum levels in various stages of DR are needed to provide a better understanding of the interaction between systemic and local inflammatory and angiogenic factors.

## Introduction

Diabetic retinopathy (DR) is one of the most debilitating complications of diabetes mellitus (DM). Although proliferative retinopathy may lead to loss of vision and blindness, diabetic macular edema (DME) is the main cause of central vision loss. The Wisconsin Epidemiological Study for Diabetic Retinopathy found DME in 3%–29% out of 1121 patients who are 30 years or older at the time of diagnosis [1] and DME is demonstrated to be responsible for blindness in 72% out of 64 blind patients due to DR [2].

DME is a multifactorial burden of DM including risk factors like duration of diabetes, insulin dependence, glycosylated hemoglobin, proteinuria, hypertension in addition to panretinal laser photocoagulation, and traction of the posterior hyaloid [3-5]. It is mainly caused by leakage of intravascular fluid from microaneurysms and abnormal retinal capillaries into the intraretinal and subretinal space due to hyperpermeability related to general blood-retina barrier (BRB) breakdown. This hyperpermeability is the ultimate result of a cascade of biochemical and cellular changes [6].

In recent years mounting evidence has emerged about the role of cytokines, inflammatory cells, growth factors, and angiogenic factors in the pathogenesis of diabetic retinopathy. These studies support the hypothesis that DR is a low grade, subclinical inflammatory disease; however the majority of them included patients with advanced DR who underwent vitrectomy and therefore lack comparison of findings with patients without DR or early DR. In these studies the levels of cytokines were determined with ELISA [7-10]. Luminex multiplex bead immunoassay, is a new technology which employs uniquely labeled fluorescent microspheres conjugated to anti-cytokine capture antibodies and allows analysis of all molecules from one sample [11]. The technology effectively allows multiple cytokines to be analyzed simultaneously from small volume samples (25–100 μl/test), reducing time, cost, and effort, making this technology a valid alternative method to ELISA. The measurements with Luminex technology also show excellent correlations with ELISA [12,13].

Our study aimed to elucidate serum levels of the most debated cytokines and chemokines including interleukin-1α (IL-1α), interleukin-6 (IL-6), interleukin-8 (IL-8), interleukin-10 (IL-10), macrophage inflammatory protein (MIP-1α), monocyte chemoattractant protein (MCP-1), epidermal growth factor (EGF), and vascular endothelial growth factor (VEGF) in subjects with DM and the healthy subjects by using the multiplex bead immunoassay. We also evaluated the relationship between serum cytokine levels and macular thickness measurements obtained with optical coherence tomography (OCT).

## Methods

In this prospective study, we enrolled 154 participants: 126 patients with type 2 DM and 28 age-matched healthy control participants. Informed consent was obtained from all participants, in accordance with the tenets of the Declaration of Helsinki, and the study was approved by the Ethics Review Board of the Selcuk University. The diabetic patients were recruited from the Retina Service, Department of Ophthalmology, Selcuk University Hospitals. Control participants were nondiabetic individuals who had visited the Ophthalmology Department at Selcuk University for routine examination or prescription of eyeglasses. Ocular exclusion criteria were intraocular surgery, intravitreal therapy, photocoagulation, trauma, vitreous hemorrhage, and retinal detachment in the preceeding six months and history of any ocular inflammatory disease like uveitis. Also excluded were DR patients who presented with DME related to posterior hyaloid traction. Systemic exclusion criteria included ischemic cerebrovascular disorders, ischemic cardiovascular disorder, hyperlipidemia, renal dysfunction (serum creatinine concentration >1.5 mg/dl), hepatic dysfunction (serum ALT >32 IU/l, serum AST>40 IU/I), hematological disease, any systemic inflammatory disease and history of malignancy. All participants underwent comprehensive ophthalmologic examination that included best corrected visual acuity, applanation tonometry, slit-lamp examination, and dilated fundus examination. Based on international clinical DR disease severity scale [14], participants were divided into four groups: 28 control participants (Group 1), 31 patients without DR (Group 2), 49 nonproliferative DR patients (Group 3), and 46 proliferative DR patients (Group 4). DM patients were classified based on the. The mean age of the control group was 64.2±8.22 years and that of the diabetic patients were 62.5±7.87 years.

The eye with the lower Snellen visual acuity of all participants was selected for the study. The macular profile was assessed with the fast macular scan protocol of the OCT-3 (Carl Zeiss Meditec, Inc., San Leandro, CA) through dilated pupil and by the same physician (B.T.O). This protocol consisted of six line scans that were 6 mm long, centered on fixation, and spaced 30 degrees apart around the circumference of a circle. The mean central subfield macular thickness of each eye was recorded for the analysis. For the correlation analysis we preferred the central subfield macular thickness instead of foveolar thickness which is a mean value, generated by the Stratus OCT software from the central A-scan thickness values of the six radial lines comprising the fast macular thickness map. In contrast the central subfield macular thickness is computed from 21 points from each of the six radial lines and was reported to provide a better representation for the central retina because of its higher reproducibility and correlation with other measurements of the central macula [15].

Blood samples were obtained from all participants by venous puncture and sent to the laboratory. The glycosylated hemoglobin (HbA1c) level was assessed. The remaning sample is centrifugated at 1000 g for 10 min after which the harvested serum was stored at −80 °C until assayed.

### Cytokine assay

IL-1α, IL-6, IL-8, IL-10, EGF, MCP-1, MIP-1α, and VEGF were measured in serum samples using Luminex multiplex bead immunoassay (Human Cytokine LINCOplex kit; LINCO Research, St. Charles, MO). Multiplex bead kits were purchased from Linco Research, Inc. (catalog number HCYTO-60K). The assay was performed according to the manufacturer’s instructions with Luminex laser based fluorescent analytical test instrumentation. Standard curves for each cytokine were generated by using the reference cytokine concentrations supplied by the manufacturer. The value “zero” represented undetectable levels of the cytokine.

### Statistical analysis

Data were analyzed using the statistical package for the social sciences (SPSS version 15.0) for Windows. Group differences between diabetics and controls were analyzed using one-way ANOVA or nonparametric Kruskal–Wallis tests, depending on normality assumptions and homogeneity of variances. The parameters showing statistically significant difference among all groups were further analyzed using Mann–Whitney-U test or Student *t*-test. The correlations between study parameters were analyzed by Spearman’s correlation test. All tests were performed at an error level of 5%.

## Results

With regard to age (p=0.24) and sex (p=0.61) distribution, no significant difference was observed between the study groups as presented in Table 1. The median value of DM duration was 10 years in Group 2, 12 years in Group 3, and 15 years in Group 4 (p<0.001; Table 1).

**Table 1:** Distribution of age, sex, and diabetes duration between groups.

** **		**Group 1 (Control) (n=28)**	**Group 2 (No retinopathy) (n=31)**	**Group 3 (Nonproliferative retinopathy) (n=49)**	**Group 4 (Proliferative retinopathy) (n=46)**	p value*
Age		64.2±8.22	63.9±9.50	63.2±8.08	60.9±6.14	0.24
Sex	Female	16 (57.14%)	17 (54.84%)	32 (65.31%)	24 (52.17%)	0.61
	Male	12 (42.86%)	14 (45.16%)	17 (34.69%)	22 (47.82%)	
Duration of diabetes mellitus (years) Median value (range) [Mean±standard deviation]		0	10.0 (1-20) [9.84±7.13]	12.0 (1-30) [12.54±6.16]	15.0 (1-30) [15.89±6.99]	<0.001

The comparison of groups enrolled in the present study revealed no statistically significant difference for age and sex. The duration of diabetes mellitus (DM) increased in the DR groups (Group3 and 4) and statistical analysis demonstrated a significant difference. The asterisk indicates the data of one-way ANOVA test.

The HbA1c values between 4.27%–6.07% were considered as normal in our laboratory. The mean HbA1c level in control group was 5.69±0.53%. The mean HbA1c level was 7.95±2.06% in Group 2, 8.10±1.61% in Group 3, and 8.53±2.11% in Group 4 (Table 2). This increase of HbA1c levels with progression of DR was found to be statistically significant (p=0.02). The median value of the visual acuity was 20/20 in Groups 1 and 2, but there was a decrease to 20/100 in Group 3 and 20/125 in Group 4. This decrease was also found to be statistically significant (p<0.001). The median value of central subfield macular thickness was estimated as 165.50 μm in Group 1, 202.5 μm in Group 2, 318 μm in Group 3, and 310 μm in Group 4. Statistical analysis showed significant differences between the groups (p<0.001) and also correlation with visual acuity (r=-0.6, p<0.001; Table 2).

**Table 2:** Comparison of HBA1c levels, visual acuity, and central subfield macular thickness values between groups.

** **	**Group 1 (Control) (n=28)**	**Group 2 (No retinopathy) (n=31)**	**Group 3 (Nonproliferative retinopathy) (n=49)**	**Group 4 (Proliferative retinopathy) (n=46)**	p value*
HbA1c (%)	5.69±0.53	7.95±2.06	8.10±1.61	8.53±2.11	0.02
Visual acuity Median values (range)	20/20 (20/32–20/20)	20/20 (20/40- 20/20)	20/100 (<20/400–20/20)	20/125 (<20/400–20/25)	<0.001
Macular thickness (μm) Median values (range)	165.5 (159–194)	202.5 (125–277)	318 (185–670)	310 (153–573)	<0.001

With regard to HbA1c levels, visual acuity and central subfield macular thickness values, there was a statistically significant difference between study groups. The asterisk indicates the data of one-way ANOVA test. Abbreviations: DR represents diabetic retinopathy.

**Figure 1:**
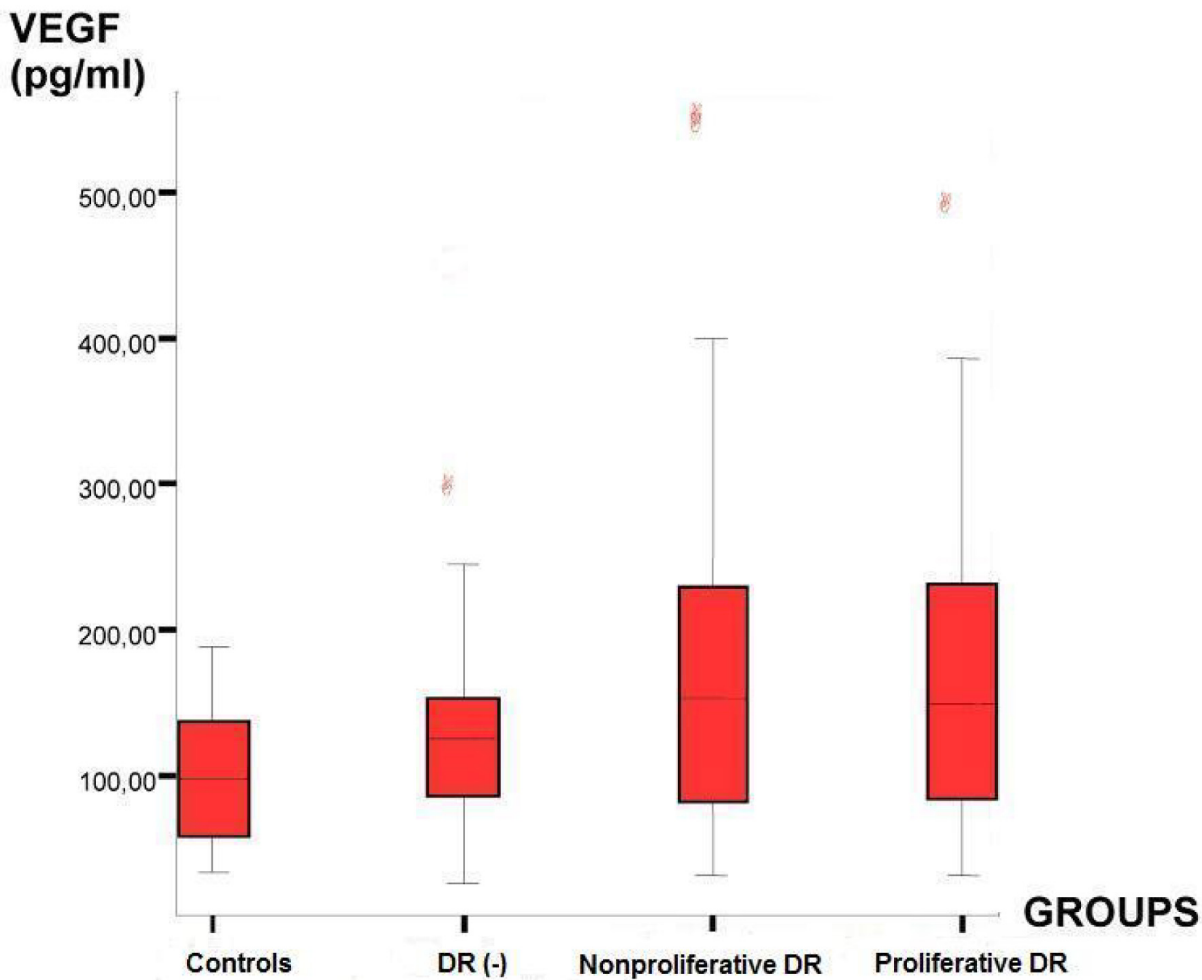
Distribution of serum VEGF concentrations within the groups. Boxplot analysis showing increased serum levels of vascular endothelial growth factor (VEGF) in patients with diabetes mellitus (DM) compared to control subjects. The median serum VEGF levels were higher in diabetic subjects with retinopathy than those without. Abbreviations: DR(-) represents patients without diabetic retinopathy.

There was significant variation between the cytokine concentrations. These values showed a remarkable overlap among the study groups. The median serum level of vascular endothelial growth factor (VEGF) was 98.20 pg/ml in the control group, 125.37 pg/ml in DM patients without DR (Group 2), 153.07 pg/ml in nonproliferative DR patients (Group 3), and 149.12 pg/ml in the proliferative DR patients (Group 4; Table 3). These higher serum VEGF levels in the DR groups as demonstrated in Figure 1 were found to be statistically significant (p=0.04). The statistical analysis revealed a significant difference in serum VEGF levels between Group 1 and Group 3 (p=0.01) and between Group 1 and Group 4 (p=0.02), but there were no statistically significant differences between Group 3 and Group 4 (p=0.87).

**Table 3:** Serum concentrations of VEGF and cytokines in the study groups

** **	** **	**Group 1 (Control) (n=28)**	**Group 2 (No retinopathy) (n=31)**	**Group 3 (Nonproliferative retinopathy) (n=49)**	**Group 4 (Proliferative retinopathy) (n=46)**	p value*
VEGF (pg/ml)	M±SD	100.47±49.66	137.29±84.45	177.07±119.51	169.88±109.12	0.04
	Mdn	98.20	125.37	153.07	149.12	
	Range	(32.46–186.93)	(24.30–445.76)	(30.03–547.74)	(31.56–487.02)	
IL-1α (pg/ml)	M±SD	13.15±32.40	5.31±19.07	3.40±12.76	99.78±484.66	0.24
	Mdn	0	0	0	0	
	Range	(0–136.17)	(0–98.6)	(0–64.15)	(0–3255.24)	
IL-6 (pg/ml)	M±SD	4.06±5.29	4.01±9.13	7.98±19.03	23.78±91.55	0.74
	Mdn	0	0	0	0	
	Range	(0–16.99)	(0–43.64)	(0–101.18)	(0–564.62)	
IL-8	M±SD	36.10±37.92	104.76±182.93	88.40±115.45	110.99±157.96	0.29
	Mdn	18.83	11.96	44.19	37.64	
	Range	(6.72–142.62)	(0–697.97)	(0–552.88)	(0–775.42)	
IL-10 (pg/ml)	M±SD	17.57±42.93	8.13±24.17	18.43±42.54	42.15±202.57	0.31
	Mdn	3.34	0	0	0	
	Range	(0–208.27)	(0–128.61)	(0–220.19)	(0–1362.73)	
MIP-1α (pg/ml)	M±SD	10.68±14.92	9.63±13.46	13.08±21.97	169.51±1061.659.15	0.4
	Mdn	4.69	0	6.91	(0–7212.84)	
	Range	(0–64.65)	(0–41.94)	(0–120.24)		
MCP-1 (pg/ml)	M±SD	154.42±58.29	193.26±103.75	192.37±78.54	311.61±488.61	0.03
	Mdn	159.0	167.95	195.56	226.59	
	Range	(44.29–243.0)	(46.42–434.28)	(0–377.19)	(96.80–3475.76)	
EGF (pg/ml)	M±SD	146.60±86.80	180.29±129.16	169.56±106.92	189.18±93.82	0.41
	Mdn	147.67	152.22	150.21	172.39	
	Range	(7.94–321.46)	(13.07–551.87)	(8.34–484.07)	(39.28–421.02)	

Serum levels of inflammatory cytokines including interleukin-1α (IL-1α), interleukin-6 (IL-6), interleukin-8 (IL-8), interleukin-10 (IL-10), macrophage inflammatory protein (MIP-1α), monocyte chemoattractant protein (MCP-1), epidermal growth factor (EGF), showed a statistically significant difference between groups only for MCP-1. The serum levels of vascular endothelial growth factor (VEGF) increase in the diabetic retinopathy (DR) groups which were found to be statistically significant as well. The asterisk indicates data of the Kruskal–Wallis variance analysis. Abbreviations: M±SD represents mean ± standard deviation, Mdn represents median.

The median levels of serum IL-1α and IL-6 were zero in all subgroups. Serum IL-1 could not be detected in 23 subjects in Group 1, 27 subjects in Group 2, 45 subjects in Group 3 and 36 subjects in Group 4. Twelve subjects in Group 1, 20 subjects in Group 2, 29 subjects in Group 3 and 26 subjects in Group 4 demonstrated undetected levels of IL-6.

The serum levels of IL-8, IL-10, MIP-1α, and EGF revealed a wide range within each group, however no statistically significant relationship was found among the subgroups. Serum IL-10 could not be detected in 11 members of group 1; the median value was 3.34 pg/ml. However, 22 subjects in group 2, 31 subjects in group 3 and 31 subjects in group 4 demonstrated a level of zero, so the median value in these groups was calculated as zero.

**Figure 2:**
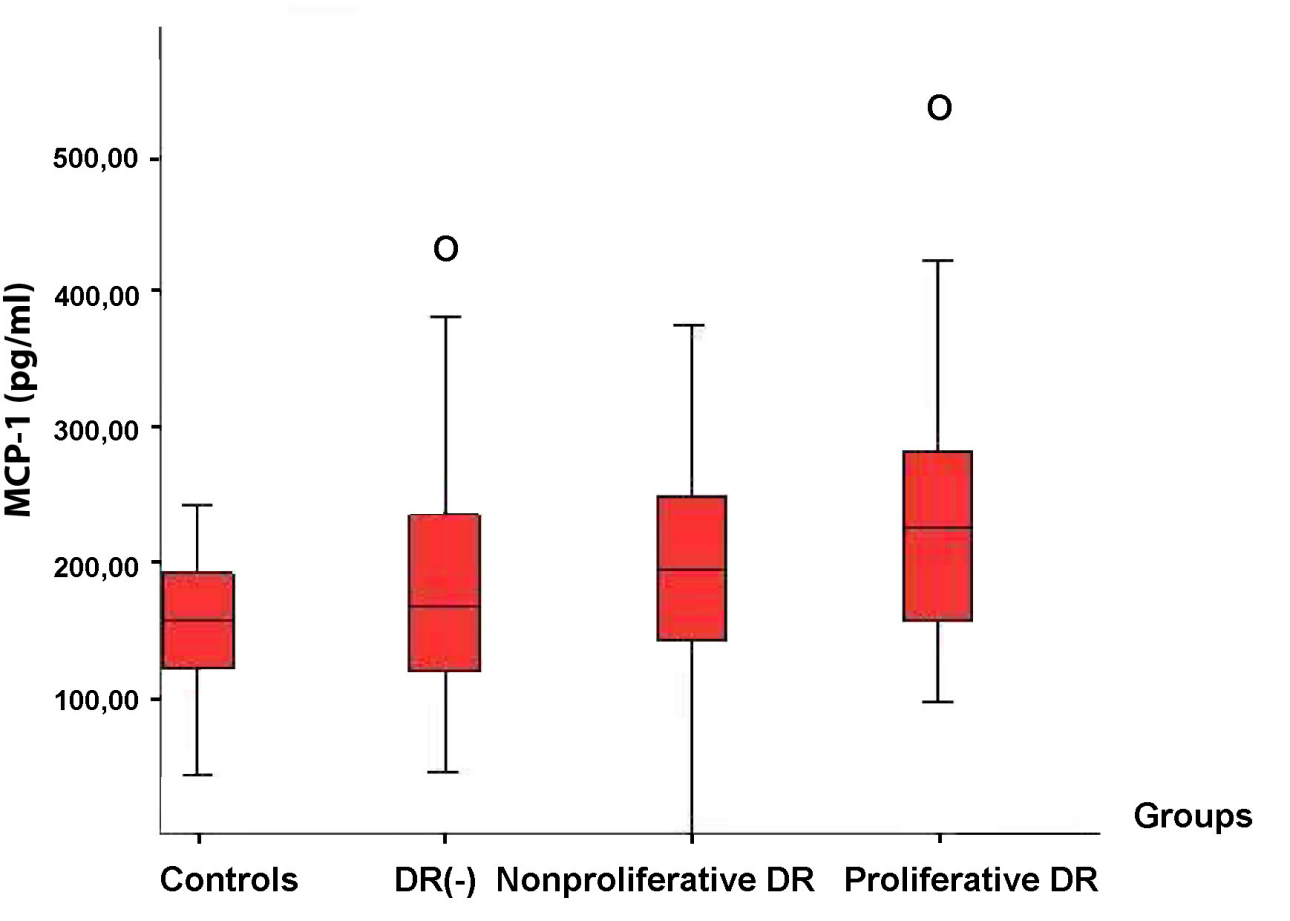
Distribution of serum MCP-1 levels within the groups. Boxplot analysis of serum monocyte chemoattractant protein (MCP-1) levels in diabetic patients demonstrated an increase with progression of diabetic retinopathy (DR). Abbreviations: DR(-) represents patients without diabetic retinopathy.

The median values of the serum MCP-1 concentrations presented a statistically significant rise with the progression of DR (p=0.03; Figure 2; Table 3). There was no statistically significant difference between Group 1 and Group 2 (p=0.31), but the difference between Group 3 and Group 4 was significant (p=0.02).

Serum cytokines and VEGF levels did not show a correlation with age and gender (Table 4). However the duration of DM was found to correlate with serum VEGF (r=0.19, p=0.02), IL-8 (r=0.27, p=0.001), MCP-1 (r=0.30, p=0.00), and EGF levels (r=0.18, p=0.03).

**Table 4:** Correlation analysis of age, gender, duration of diabetes mellitus, HbA1c levels and macular thickness.

** **	**Age**		**Gender**		**Duration of DM**		**HbA1c**		**Macular thickness**	
	**Coefficient p**		**Coefficient p**		**Coefficient p**		**Coefficient p**		**Coefficient p**	
VEGF	−0.01	0.89	−0.06	0.44	0.19	0.03	0.04	0.63	0.06	0.5
IL-1α	0.03	0.73	0	0.95	−0.05	0.51	−0.08	0.36	0.06	0.49
IL-6	−0.05	0.55	−0.11	0.16	0.03	0.7	−0.03	0.7	0	1
IL-8	0.1	0.22	−0.02	0.81	0.27	0	−0.03	0.77	0.13	0.16
IL-10	0.14	0.08	0	0.98	−0.04	0.63	−0.25	0	0.11	0.21
MIP-1α	0.1	0.23	−0.06	0.44	0.13	0.11	−0.08	0.37	0.14	0.13
MCP-1	−0.06	0.46	0.02	0.82	0.3	0	0.18	0.05	0.15	0.12
EGF	−0.03	0.67	0.07	0.39	0.18	0.03	−0.05	0.59	−0.01	0.91

The Spearman’s correlation analysis of the inflammatory cytokines including interleukin-1α (IL-1α), interleukin-6 (IL-6), interleukin-8 (IL-8), interleukin-10 (IL-10), macrophage inflammatory protein (MIP-1α), monocyte chemoattractant protein (MCP-1), epidermal growth factor (EGF), and vascular endothelial growth factor (VEGF), with age, gender and macular thickness showed no correlation. Duration of diabetes mellitus (DM) correlated with serum VEGF, IL-8, MCP-1, and EGF levels and HbA1c levels correlated with IL-10 and MCP-1 levels. The coefficients were calculated from the Spearman’s correlation test p<0.05.

Serum HbA1c levels revealed a significant correlation with IL-10 and MCP-1 levels (r=-0.25 p=0.005 and r=0.18 p=0.05 respectively), however there was no correlation with serum VEGF levels (r=0.04 p=0.63). The serum VEGF levels were estimated to be correlated with IL-6, IL-8, MIP-1α, MCP-1 and EGF levels (p<0.05 for all) and MCP-1 levels were found to be correlated with HbA1c and VEGF as mentioned above and with IL-8, MIP and EGF levels (p<0.001 for all). A correlation between the amount of macular edema quantified with the measurement of the central subfield macular thickness and the serum levels of the studied cytokines and VEGF was lacking according to the correlation analysis (p>0.05; Table 4).

## Discussion

Despite advances in ophthalmological care, DR remains a major cause of preventable blindness [16]. Current therapeutic strategies indicate that a better understanding of the pathogenesis is crucial for an improved management of DR. It has been recognized that the metabolic control, reflected by the blood glucose level and glycosylated hemoglobin value, is an important factor for the onset and progression of DR. Nevertheless the precise pathogenic mechanism of DR is unclear already [17].

DME, the primary clinical feature associated with visual impairment, is known to occur from the leakage of plasma into the central retina. This results in stretching and distortion of neurons and leads to reversible reduction in visual acuity. Over time, the perturbed neurons die, resulting in permanent visual loss [18]. The leakage may be focal or diffuse. Focal leakage might occur due to leakage from microaneurysms. These microaneurysms resulted from the retinal vessel wall weakening and impairment of the BRB due to hyperglycemia induced pericyte death. In contrast diffuse leakage is related to the microscopic damage of retinal vessels described as BRB breakdown and increased permeability factors like IL-6 and VEGF [5].

DR displays all microscopic signs of inflammation such as vasodilatation, altered flow, fluid exudation and leukocyte migration. Therefore chronic low grade inflammation seems to be an inciting and final common pathway leading to DR. (8). Alterations in serum or vitreous levels of many inflammatory cytokines like IL-6, IL-8, IL-10, and VEGF also supports the role of inflammation in DR [10,19-21].

VEGF is the most attractive candidate for stimulating new vessel formation and vascular hyperpermeability in DR. It is a mitogen for endothelial cells, and its expression both in vivo and in vitro can be induced by hypoxia [22,23]. Retinal hypoxia-induced inflammation and increased expression of VEGF has been implicated in the pathogenesis of DME [24]. The pivotal role of VEGF in DME is further supported with the studies that showed regression of DME after the intravitreal injection of anti-VEGF drugs [25].

In the literature, the association of serum VEGF levels with DR are conflicting. Most of the studies evaluating both vitreous and serum VEGF levels have found increased VEGF levels in diabetics compared to controls [26-28]. In our study, serum VEGF levels were also significantly higher in the diabetic group compared to controls, with the highest values observed in DR group. However, there was no significant difference between the nonproliferative DR group (Group 3) and the proliferative DR group (Group 4; p=0.87), which may be an explanation for the lack of correlation with HbA1c levels (Table 3). Meleth et al. [26] found also no significant difference in serum VEGF, IL-6, and IL-8 levels among subjects with less severe and severe DR. In contrast Cavusoglu et al. [26] detected progressively increasing serum levels of VEGF in DR and correlation with the stage of retinopathy and HbA1c levels. The high levels of VEGF in the serum of patients with DR both in our study and that of Cavusoglu may support its role in development of retinopathy.

IL-6 is a multifunctional cytokine that indirectly causes an increase in vascular permeability and neovascularization by inducing the expression of VEGF [29]. It can also directly increase endothelial cell permeability in vitro by rearranging actin filaments and by changing the shape of endothelial cells [30]. Serum levels of IL-6 and TNF- α are proposed to be predictor of proliferative retinopathy development [31]. IL-8 has been recognized as a potent chemoattractant activator of neutrophils and T lymphocytes but not monocytes [32]. In the study of Murugeswari et al. [33], the vitreous levels of IL-6, IL-8, MCP-1 and VEGFs were significantly higher in proliferative DR and serum levels of IL-8, VEGF and MCP-1 were below the vitreous levels, which elucidate a local inflammatory process in DR. In the study Hernandez et al. [10], vitreous levels of IL-8 and MCP-1 were found to be increased in proliferative DR. Meleth et al. [28] could not find any difference in serum IL-6 and IL-8 levels between less severe and severe DR and the percentage of subjects with undetectable serum levels of IL-8 and IL-6 .was over 80%. In the study of Doganay et al. [20], the levels of serum IL-6 were below the detection limits of the assay in all patients with DM and controls and IL-8 levels were found to be highest in proliferative DR group. In our study, serum IL-6 could not be detected in 58% of DR group and 43% of control subjects. Both serum IL-6 and IL-8 did not different among the study groups.

IL-1α is a proinflammatory cytokine that has pleiotrophic and overlapping functions and is neuroprotective in CNS [34]. In this study, serum IL-1α levels did not differ between healthy subjects and diabetic patients, which might be due to the undetectable serum levels of IL-1α in the majority of subjects. In the study of Doganay et al. [20], the levels of IL-1β were below the detection levels in all DR patients and healthy controls.

Another cytokine evaluated in our study was IL-10, an anti-inflammatory cytokine produced primarily by monocytes and macrophages [35]. Lee et al. [31] found that higher serum IL-10 levels are related to lower risk of DR in DM patients. Supporting this finding, our study noted higher serum IL-10 levels in control participants compared to diabetic patients. However, a statistically proven relationship was lacking (p=0.31).

Chemokines are small molecular weight proteins that guide the migration of responsive cells. Cells attracted by chemokines shows chemoattraction in addition to leukocyte activation. They are categorized into four subgroups: CXC, CC, C, and CX3X. MIP-1α and MCP-1 are members of the CC chemokines [36].

MCP-1 is a chemokine that recruits immune cells such as monocytes and lymphocytes [37]. It is produced by retinal endothelial cells and has been implicated in leukostasis of the hypoxic retina [28,38]. Hyperglycemia has also been shown to increase the expression of MCP-1 by vascular endothelial cells [39]. A positive regulatory feedback loop between VEGF and MCP-1 expression by vascular endothelial cells has been also suggested [9]. Previous data proposed that MCP-1 is a potential angiogenic factor in the proliferative phase of DR [10,21,40]. Mitamura et al. [40] and Hernández et al. [10] found a significant association between MCP-1 levels in the vitreous and the degree of proliferation in DR. Maier et al. [21] showed a positive correlation of HbA1c levels with vitreous MCP-1 levels, but could not find a correlation between MCP-1 levels and stage of DR. Our data demonstrate correlation of MCP-1 with the progression of DR and also with HbA1c levels, VEGF, IL-8, and MIP-1α.

MIP-1α is produced by macrophages and activates human granulocytes such as neutrophils, eosinophils, basophils, and monocytes. MIP-1α also induces the release of proinflammatory interleukins such as IL-1 or IL-6 [41]. In the mouse model, MIP-1α has been identified as a potent inducer of retinal neovascularization [42]. Hanifi-Moghaddam et al. [43] observed an upregulation in serum levels of MIP-1α in patients with type 1 DM. In contrast,in the study of Capeans et al. [44] MIP-1α levels have been noted to be below the detection levels in both diabetics and controls. Our study showed an upregulation of MIP-1α in the diabetic group compared to controls. This finding was not considered significant statistically. However, we found a significant correlation with IL-6, IL-8, IL-10, and VEGF supporting its role in retinal neovascularization.

Several growth factors have been implicated in the development of preretinal fibrovascular membranes in proliferative DR [45,46]. One of these factors is EGF, which is known to be a potent migrator as well as a cell migration and differentiation modulator. It is still a matter of debate as to whether EGF is of systemic origin or is derived locally from retina [47]. The insignificant difference of serum EGF levels between control subjects and diabetic patients with different stages of DR found in our study is in accordance with the study of Patel et al. [47] and infers local production of EGF in DR.

To the best of our knowledge, our study is the first that evaluated the correlation of serum cytokines and VEGF levels with macular thickness of diabetic patients with DME and found no correlation (Table 4). Similar to our study, Petrovic et al. [27] found no differences in serum VEGF levels between DM patients with and without macular edema and with and without active neovascularization. But the presence of macular edema was based only on clinical examination in their study. Patel et al. [8] evaluated the vitreous and aqueous levels of VEGF and detected the highest vitreous levels in the NPDR group followed by the proliferative DR group. They additionally divided subjects with DME into two groups according to the macular profile: Group 1 with posterior hyaloid traction and Group 2 with diffuse low elevation of macula. The vitreous and aqueous VEGF levels in Group 2 were higher compared to Group 1, which supports the hyperpermeability hypothesis in this group of DME. Funatsu et al. [48] also demonstrated a correlation between vitreous VEGF, ICAM, IL-6, MCP-1, and PEDF levels with DME severity. Regarding these findings, the lack of correlation between macular thickness and serum VEGF levels in our study might indicate the importance of local VEGF production in DME [8,48]. However we could not compare the serum levels of the abovementioned cytokines and VEGF with vitreous levels, since none of the subjects consented an invasive procedure like vitreous tap.

Another reason for the lack of correlation between serum cytokine levels and VEGF with DME might be the effect of other microvascular diabetic complications on serum levels. Increasing evidence highlights the importance of inflammatory mechanisms in the development and progression of other microvascular diabetic complications such as neuropathy and nephropathy [49,50]. Therefore we excluded diabetic subjects with nephropathy to evaluate directly the relationship between serum cytokines and DME. However, it did not completely eliminate the effect of other organs on serum levels of cytokines and VEGF, since it is recognized that features of inflammation are displayed years before the onset of complications.

In conclusion, we suggest that increased serum levels of VEGF and MCP-1 may act as a key regulator of DR and provide a potential tool for risk assessment of DR. Further studies comparing both vitreous and serum levels in various stages of DR are needed to provide a better understanding of the interaction between systemic and ocular inflammatory and angiogenic factors
